# Queries as to Rhus and Other Poisonings

**Published:** 1888-07

**Authors:** Frederick Hooker

**Affiliations:** Syracuse, N. Y.


### QUERIES AS TO RHUS AND OTHER POISONINGS.

EDITOR HOMŒOPATHIC PHYSICIAN: In replying to Dr. Farley's letter, in the June number of THE HOMŒOPATHIC PHYSICIAN, I wish to ask the Doctor a few questions.

*First.*—Is the disappearance of the immediate symptoms a sign of *cure?*

*Second.*—If so, why do the symptoms recur, in many instances at the same time every year, without a fresh exposure?

Dr. Seward cited a case of eighteen years' standing.

*Third.*—If Dr. F. was cured, why is he still so extremely susceptible to the poison?

*Fourth.*—Does the Doctor deny that the poison may be carried to the face and genital organs by the unwashed hands, as well as by systemic infection?

FREDERICK HOOKER, M. D.

SYRACUSE, N. Y., June 4th, 1888.

EDITORS HOMŒOPATHIC PHYSICIAN: If Rhus tox. C. M. or D. M. or any other high potency WILL cure a case of "Rhus poisoning," why will NOT a high potency of Crotalus cure the poisonous effects of the rattlesnake bite, a high potency of Arsenic or Strychnia cases of poisoning by these respective drugs, and so on "ad finem"?

Is it possible that the cases above mentioned are not analogous, even under the guidance of the isopathic *law?**

Confusion utter holds the mind a prey.
When, though convinced,
Opinions change from day to day.

* There is no isopathic *law* that we have ever heard of; the only law of *cure* known is that of the similars, under which one prescribes for each case the remedy most similar to the symptoms manifested. It is doubtless true that a high potency will frequently remove the symptoms produced by the crude drug, but in such cases the remedy will *always* be found to be well indicated by the symptoms. Homœopathy needs no theories, nor any guessing; it is the science of medicine based upon an immutable *law*.—EDITORS H. P.

